# Sensitively and Selectively Detect Biothiols by Using Fluorescence Method and Resonance Light Scattering Technique Simultaneously

**DOI:** 10.3390/molecules24224136

**Published:** 2019-11-15

**Authors:** Yanping Shi, Chao Sun, Xiaoqi Gao, Wei Zhao, Nan Zhou

**Affiliations:** 1 Department of Chemistry, Northeast Agricultural University, Harbin 150025, China; shiyanping160@aliyun.com (Y.S.); sscc961027@163.com (C.S.); 18840614060@163.com (X.G.); 2 Department of Physiology, Hei Longjiang University of Chinese Medicine, Harbin 150040, China; paradise1100@sina.com

**Keywords:** carbon quantum dots, RLS technique, IFE, biothiols, Ag^+^ complex

## Abstract

In this paper, we designed a new quantitative and qualitive detection method for biothiols by using fluorescence method and resonance light scattering (RLS) technique. Nitrogen doped carbon quantum dots (C/N-dots) were obtained from tartaric acid and ethylenediamine by hydrothermal method, and then their morphology and optical properties were characterized by different techniques. A detection system consisting of C/N-dots and Ag^+^ complex was established. In this system, C/N-dots possessed the photoluminescent property and the Ag^+^ complex owned the RLS property, so, by combining the two luminescent properties to achieve complementary advantages, we could detect biothiols and solve the problem of distinguishing between Cys and GSH. Additionally, we optimized detecting conditions and investigated the detection mechanism of fluorescence quenching and RLS detecting. Results showed that the analytical response of fluorescence was linear in the range 0–140 μM and the detection limit (LOD) was calculated to be 6.6 μM for Cys, and the addition of GSH had no effect on fluorescence. RLS response ranges were 0–167 μM for Cys and 0–200 μM for GSH, with LOD down to 64 nM and 74 nM, respectively. Furthermore, the probe was successfully used for detecting Cys in fetal bovine serum (FBS) samples by fluorescence method, and also, by RLS technique, the content of GSH in FBS samples was detected.

## 1. Introduction

Biological aminothiols, also called biothiols [1], mainly contain cysteine (Cys) and glutathione (GSH). Cysteine, a kind of small molecular substance, possessing vital physiological functions in living organisms. Generally speaking, its normal concentration is maintained at the level of micromole, and normal Cys concentrations in blood plasma range from 135 to 300 μM [2]. Abnormal levels of them will cause a variety of diseases, such as skin damage, liver damage, cardiovascular disease, muscle loss, Alzheimer’s disease, etc. [3,4,5]. GSH, a thiol-containing tripeptide, has a significant effect on the maintenance of genetic regulation [6], cellular signal transduction and reversible redox reactions [7,8], such as intracellular redox states. In addition, it not only has an essential role in the human metabolic process, but also plays a role in detoxification in plants [9], such as reactive oxygen species (ROS) detoxification and the detoxification of organic xenobiotics. Due to their various fundamental functions in cellular systems [10,11], their content change is closely related to early diagnosis of diseases. Compared with other amino acids (AA), Cys and GSH both have a sulfhydryl group. Therefore, it is easy to distinguish them from other AA by detecting the sulfhydryl group. However, making a distinction between Cys and GSH is still a great challenge, due to their similarity in structure [12]. Although some organic fluorophores, which are sensitive to sulfhydryl groups, can be used to discriminate biothiols [13,14], the complicated preparation process limits their application. In summary, making a distinction between Cys and GSH rapidly and sensitively is sought for. At present, plenty of detection techniques have been explored to detect biothiols, including high performance liquid chromatography (HPLC) [15], electrochemistry [16], gas chromatography (GC) [17,18], electronic nose (EN) [19,20], electronic tongue (ET) [20], chemiluminescence [21], mass-spectrometry [22,23], etc. However, most methods are complex, expensive, and insensitive for detection, and, more importantly, they cannot distinguish them separately. Therefore, identifying one single sulfhydryl species remains a challenge [24]. In recent years, due to their unique fluorescent properties and wide application in biomedicine [25,26], carbon quantum dots have attracted researchers’ increasing attention. Switching method is the most commonly used fluorescent method for detecting biothiols, for example, when sulphide metal, such as Hg^2+^, is added into a C/N-dots solution, Hg^2+^ ions are easily bound onto the surface of C/N-dots by coordination interactions [27], which can cause significant fluorescence quenching. When biothiol is added into the above solution, the strong interaction between Hg^2+^ and -SH can form an Hg-S bond, which can cause the separation of Hg^2+^ from the surface of C/N-dots, and so the fluorescence intensity will recover. However, there are many problems in switching methods, such as poor selectivity [28], and substances that can combine with Hg^2+^ will have an influence on detection. As a spectral method, the RLS technique has attracted great attention, and was successfully applied to detecting various analytes, such as glucose [29], protein [30], DNA [31], etc. In addition, the requirements for sensitivity, universality and simplicity of detection were all satisfied [32]. In recent years, there have been great applications for the detection of biothiols in the field of RLS technique [33]. Gold and silver nanoparticles are the most popular nanomaterials for biothiol sensing [34]; compared with gold nanoparticles, the research on RLS of silver nanoparticles is very limited. It is worth noting that biothiols possess a strong binding capacity with Ag^+^ [35], and so it is easy to capture Ag^+^. Therefore, we used RLS to study the interaction between biothiols and an Ag^+^ complex. In summary, the fluorescence quenching method and the RLS method depend on different detection mechanisms, and each has its own advantages, e.g., the RLS method has a high sensitivity, while the fluorescence quenching method is more suitable for selective detection and has a higher stability. Both fluorescence quenching and RLS methods face the same problem in the detection of biothiols in organisms: it is difficult to ensure sensitivity, linearity and selectivity at the same time, especially when distinguishing Cys and GSH. If we could combine the two methods, the detection effect could be improved.

In this paper, a selective and sensitive method for the determination of Cys and GSH using a C/N-dots–Ag^+^ complex system as a probe was established. The C/N-dots–Ag^+^ complex system was built by adding AgNO_3_ solution (0.01M) into C/N-dots phosphate buffer solution, and, under these circumstances, C/N-dots obtained the photoluminescent property and the Ag^+^ complex possessed the RLS property. Combining the above luminescent properties, we realized two different spectral detecting methods in the same system, with the same equipment. Optimal testing conditions for Cys and GSH detection were investigated and the corresponding mechanisms were proposed. The addition of Cys made it adsorb onto the surface of the Ag^+^ complex, which led to the enhancement of absorbance at 280–350 nm in UV-vis absorption spectrum, and this effect quenched the fluorescence of the sensor through inner filter effect (IFE). Moreover, it reduced the intensity of RLS by changing the surface structure of the complex. However, the addition of GSH could cause RLS intensity to drop sharply, by promoting the dissolve of C/N-dots–Ag^+^ complex without affecting the fluorescence. Therefore, fluorescence quenching method is suitable for the selective detection of Cys and GSH. A good linear range and LOD can be obtained when the sensitivity of Cys and GSH is detected by the effect of RLS. Thus, it can be seen that the two approaches possessed complementary effects in terms of sensitivity and selectivity. This sensor was successfully employed for the detection of Cys and GSH in FBS sample.

## 2. Results and Discussions

### 2.1. Synthesis and Optical Properties of C/N-Dots

**Figure 1:**
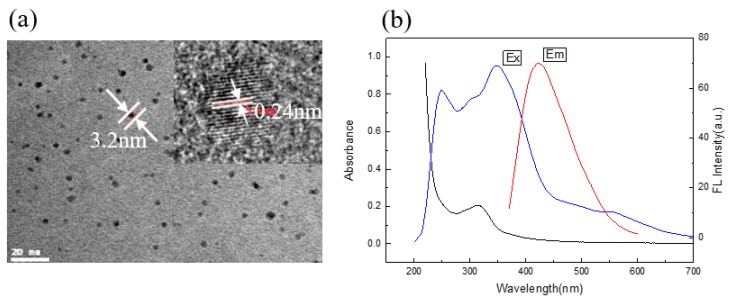
(**a**) The TEM images of C/N-dots; (**b**) UV-vis absorption (black line) and excitation (blue line) and emission (red line) spectra of the aqueous dispersion of the C/N-dots.

Tartaric is a polyhydroxyl binary carboxylic acid, and ethylenediamine contains nitrogen. Both of them are environmentally friendly [36,37] raw materials. Brown and well-distributed C/N-dots solution was obtained with a simple hydrothermal treatment of raw materials at 180 °C for 5 h. TEM characterization results clearly revealed that the C/N-dots were spherical dots and well dispersed from each other, with a diameter of about 3.2 nm. Furthermore, HRTEM was used to investigate the subtle structure of as-prepared C/N-dots. Figure 1a showed that the lattice spacing was 0.24 nm, which denoted the graphite properties of C/N-dots [38,39]. The UV-visible absorption and fluorescence spectra of C/N-dots were shown in Figure 1b, on which an obvious absorption peak at around 312 nm from the resultant C/N-dots was seen, and resulting from n-π* transitions [40,41]. The maximum excitation wavelength of C/N-dots was observed at 250 nm and 345 nm, respectively, and the maximum emission of C/N-dots was focused at 430 nm when the excited wavelength was 345 nm.

### 2.2. Characterization of C/N-Dots

**Figure 2:**
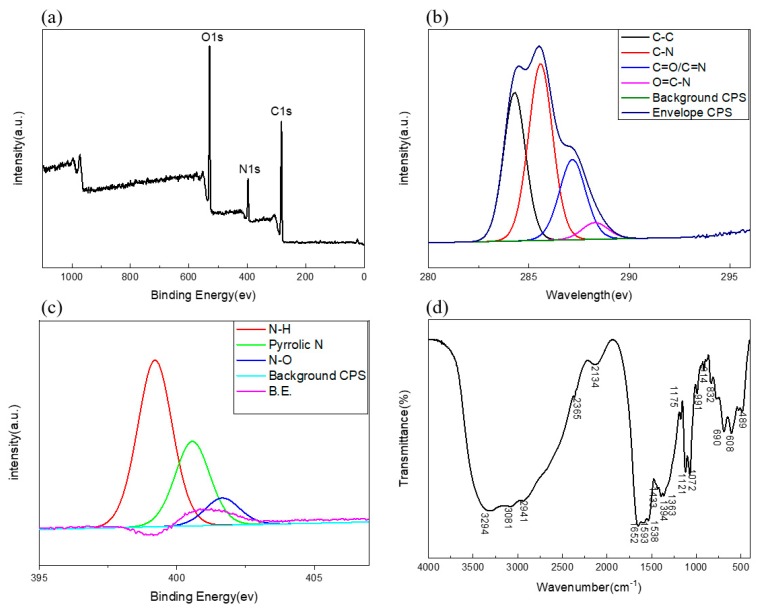
(**a**) The full-scale XPS spectrum of C/N-dots. High-resolution C1s (**b**) N1s (**c**) XPS spectra of C/N-dots. (**d**) FTIR spectrum of C/N-dots.

FTIR and XPS were used to study the surface functional groups and element states of C/N-dots. Results were shown in Figure 2a. Three typical peaks, at 283 eV, 397 eV, and 529 eV were shown in the full scan XPS, which were attributed to C1s, N1s, and O1s, respectively. The results demonstrated that C/N-dots were composed of carbon (64.25%), nitrogen (10.17%) and oxygen (25.60%). The content of N was higher than that of carbon dots in the general literature [42,43,44], which corresponded to the self–surface passivation of carbon dots. As shown in Figure 2b, the C_1s_ spectrum displayed four main peaks at 284.3 eV, 285.6 eV, 287.15 eV, 288.3 eV, which were associated with C–C, C–N [45], C=O/C=N, and O=C–N [46], respectively. As exhibited in Figure 2c, for the N_1s_ spectrum, there were three binding energy peaks, at 399.2, 400.55 and 401.65 eV, that probably resulted from N–H, pyrrolic N and N–O. These results proved the successful doping of N in the resultant C/N-dots. The functional groups of C/N-dots were characterized by FTIR spectroscopy and the spectrum was given in Figure 2d. The peak at 2941–3294 cm^−1^ is associated with stretching vibrations of N–H. The bending vibrations of N–H, which appeared at approximately 1433 cm^−1^ and peaked at about 1652 cm^−1^ arose from C=O [47], while those at around 1652 and 1433 cm^−1^ were assigned to the neighbouring conjugated structures of C=O and C–N, respectively [48]. The peak at 1593 cm^−1^ could indicate the presence of C=N [49]. The results of XPS were in agreement with previous FT-IR data.

### 2.3. Analysis of C/N-Dots

**Figure 3:**
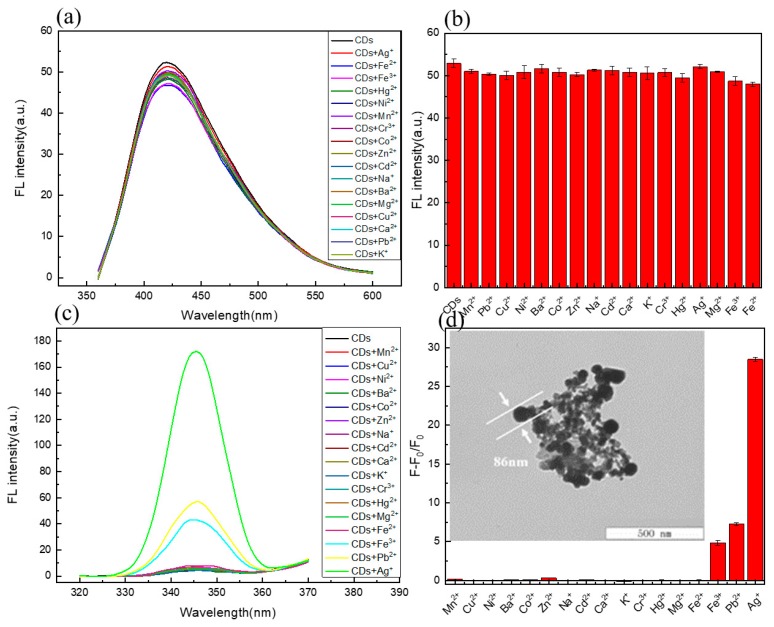
(**a**) Response of FL intensity in C/N-dots aqueous with the addition of different metal ions; (**b**) Effects of metal ions on the FL intensity of C/N-dots; (**c**) Response of RLS intensity with the addition of different metals ions; (**d**) Effects of metal ions on the RLS intensity of C/N-dots. F and F_0_ correspond to the FL intensities of the C/N-dots–Ag^+^ complex system with and without metal ions (33.3 μM), respectively.

To investigate the selectivity [36,50] of C/N-dots to metal ions, several fluorescence spectra were exhibited. The fluorescence responses of C/N-dots with the addition of various metal ions were explored. The concentration of the metal ions was 33.3 μM, and the changes in the fluorescent intensity of both the emission peak and scattering peak were collected at the same time. As shown in Figure 3a,c, fluorescent (FL) intensity had no obvious sensitivity to metal ions, however, the scattering peak increased sharply with the addition of Ag^+^, which was related to the unique optical property of Ag^+^. TEM results showed that the addition of AgNO_3_ led to the formation of an Ag^+^ complex in the system with a size of about 86 nm, which was the reason for the strong scattering peak. Although lead and iron ions can cause scattering, this is not obvious. The fluorescence response of other metal ions was shown in Figure 3b, and the corresponding RLS changes were shown in Figure 3d. It was clearly demonstrated that the as-prepared C/N-dots were insensitive to almost 17 metal ions at the range of 345–600 nm, implying that C/N-dots could shield the interference of metal ions. However, when the range of the scattering peak was captured, the response of silver ions was found. Meanwhile, biothiols possessed a strong complexing ability to silver ions, which could be used to detect biothiols.

**Figure 4:**
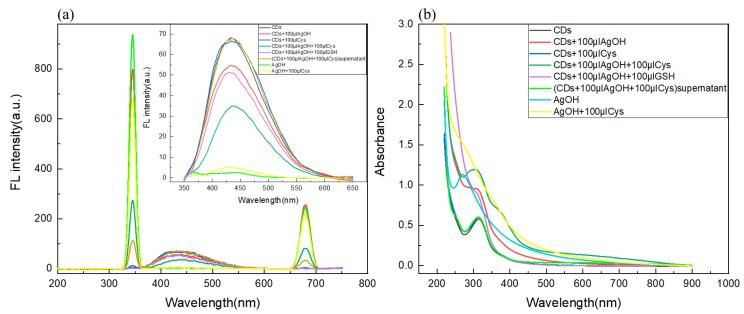
(**a**) The RLS intensity of the C/N-dots–Ag^+^ complex system with the addition of Cys and GSH; the insets corresponded to FL changes; (**b**) The corresponding UV-Vis absorption spectra of the above solution.

In order to investigate the feasibility of using a C/N-dots–Ag^+^ complex as a probe to detect Cys and GSH, we studied the corresponding variations in fluorescence and UV-vis spectra. The results are shown in Figure 4. A certain number of C/N-dots were added into phosphate buffer, then 100 μL (0.01 mol/L) AgNO_3_ was added into the above solution to construct the C/N-dots–Ag^+^ complex system. Cys and GSH were added into this system. Results showed that fluorescent intensity possessed obvious quenching in the emission peak with Cys addition, while the corresponding UV-vis absorption spectrum increased significantly at 280–350 nm, and its absorption band overlapped with the absorption band of C/N-dots. The Ag^+^ complex was separated by centrifugation and a supernatant was obtained for fluorescent detection. As shown in Figure 4a, UV-vis and fluorescence absorption spectrum of C/N-dots recovered, indicating that Cys adsorbed onto the surface of Ag^+^ complex, which led to the enhancement of absorbance at 280–350 nm in the UV-vis absorption spectrum and the fluorescence quenching of the sensor through inner filter effect (IFE). There was no obvious change with the addition of GSH. Therefore, the C/N-dots–Ag^+^ complex system obtained the ability to distinguish between Cys and GSH, and quantitatively analyze Cys. As shown in Figure 4a, the Ag^+^ complex could cause the enhancement of RLS in a C/N-dots–Ag^+^ complex system. Adding equal amounts of Cys and GSH into the system could both lead to a reduction in RLS. However, the decrease with the addition GSH was greater than with Cys, indicating that the system was more sensitive to GSH. Furthermore, the addition of GSH made the solution clear, indicating that GSH played a role in promoting the dissolution of the Ag^+^ complex, and that was why RLS intensity reduced. However, the addition of Cys could not make the Ag^+^ complex dissolve and RLS disappeared after centrifuged. This proved that Cys could adsorb onto the surface of the Ag^+^ complex, and thus modify the surface properties, which led to a reduction in RLS intensity. Its mechanism differed from GSH. In sum, a C/N-dots–Ag^+^ complex could detect both Cys and GSH by RLS, and its sensitivity was higher than fluorescence methods, but it could not discriminate them. Therefore, the two spectroscopic methods could be combined to make a distinction between Cys and GSH and also improve the sensitivity of detection.

**Figure 5:**
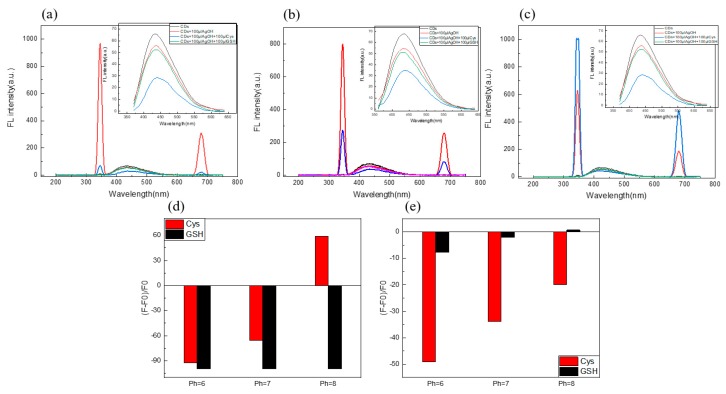
(**a**) The effect of pH (6.0 (**a**), 7.0 (**b**), 8.0 (**c**), Phosphate buffer solution) on the intensity of both the RLS and FL of the C/N-dots–Ag^+^ complex system by adding Cys and GSH; the insets corresponded to FL changes; the corresponding columnar changes of fluorescence intensity (**d**) and RLS intensity (**e**).

In order to obtain the optimal detection conditions, the effects of pH on the fluorescence intensity of Cys, and GSH on the C/N-dots–Ag^+^ complex system, were investigated in the subsequent experiment. Firstly, the quenching rate of Cys was studied, as shown in Figure 5: at pH = 6, the quenching rate was −49.07%; at pH = 7 the quenching rate was −33.76%; at pH = 8, the quenching rate was −19.91%; and, as for GSH, quenching rates were −7.7%, −2.12% and −2.79%, respectively. Therefore, we excluded pH = 8. Regarding the scattering peak, the quenching rates for Cys were −92.69%, −65.55% and 59.13%, respectively, and for GSH, they were −99.81%, −99.82% and −99.72%, respectively. No obvious change was found in detecting GSH by scattering peak. Because there was only a slight difference in detecting Cys and GSH at pH = 6, PH = 7 would be the best choice. In addition, considering the application of the probe to the serum sample, the best choice for this experiment was pH = 7.

### 2.4. Selectivity and Interference Measurements

**Figure 6:**
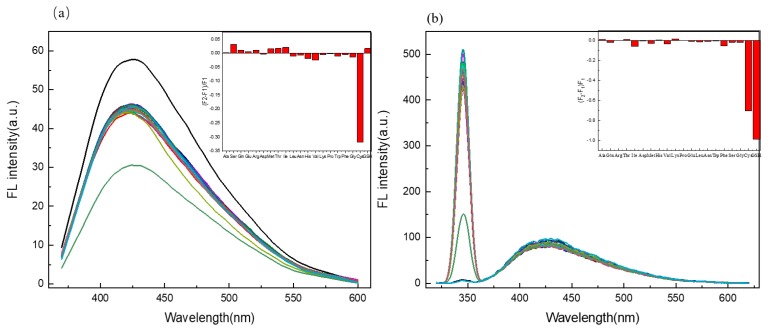
(**a**) The selectivity of the C/N-dots–Ag^+^ complex system to different AA (100 μM), the insets show (F_2_-F_1_/F_1_) of the C/N-dots–Ag^+^ complex system with the addition of different AAs; (**b**) The selectivity of RLS to the C/N-dots–Ag^+^ system with the addition of various kinds of AA (100 μM), F_2_ and F_1_ refer to the intensity of the C/N-dots–Ag^+^ complex system with and without AAs (100 μM), respectively.

To evaluate the selectivity of the sensing system towards AA, twenty AAs were added to measure the variation of fluorescence intensity of the probe system, with each at 333 μM under the same measuring condition. It can be easily seen from Figure 6a that the presence of other AA showed no obvious interfering effect on the fluorescence of the C/N-dots–Ag^+^ complex system, except for the addition of Cys, which resulted in the fluorescence quenching. The change in the fluorescence intensity of the C/N-dots–Ag^+^ complex system was described using F_2_-F_1_/F_1_. Meanwhile, regarding the scattering detection, as shown in Figure 6b, the C/N-dots–Ag^+^ complex system showed selectivity for Cys and GSH compared with the other AA. This suggests that the bonding strength of the C/N-dots–Ag^+^ system was strong enough to compete against all AAs, except GSH.

### 2.5. Linear Relationship

**Figure 7:**
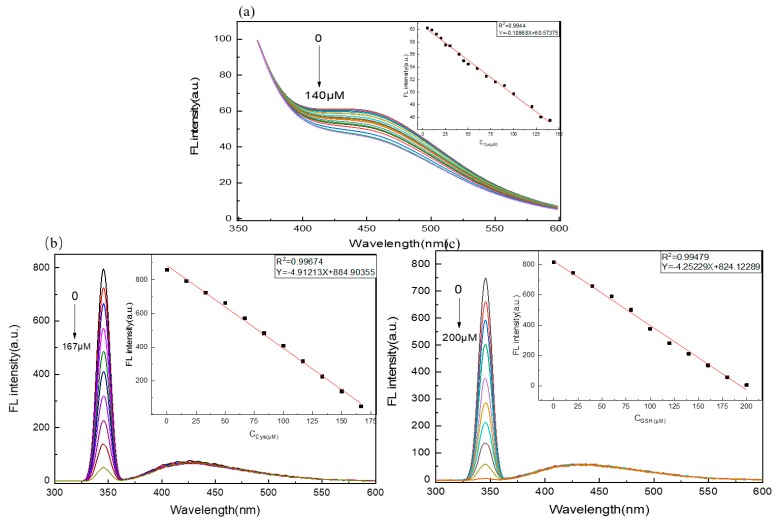
(**a**) The fluorescence spectra of C/N-dots with different concentration of Cys. The insets in (a) illustrate FL intensity against concentration of Cys. The RLS spectra in the presence of different concentration of Cys (**b**) and GSH (**c**) respectively. The insets in (**b**) and (**c**) illustrate FL intensity of scattering peak against concentration Cys of and GSH, respectively.

Under the optimized conditions, various concentrations of Cys were added to the C/N-dots–Ag+ complex system. The fluorescence intensity was collected to evaluate the sensitivity and linearity of the C/N-dots–Ag^+^ complex system sensor for detecting Cys. As displayed in Figure 7a, the fluorescence intensity of the C/N-dots–Ag^+^ complex system decreased gradually at around 430 nm, along with the increased Cys. With a linear correlation of R^2^ = 0.9944, the linear relationship was expressed as Y = −0.10968X + 60.57375 in the concentration range 0–140 μM, where Y was the fluorescence intensity of the C/N-dots–Ag+ complex system in the presence of Cys, and X was Cys concentration. The LOD was calculated to be 6.6 μM. Meanwhile, we found that the intensity of the scattering peak linearly varied with the increase in Cys. As shown in Figure 7b, the RLS intensities of the C/N-dots–Ag^+^ complex system decreased gradually with the increase in Cys. With a wide concentration range, from 0 to 167 μM, the probe exhibited a good linear relationship toward Cys at 345 nm scattering peak, and the equation could be expressed as Y = −4.91213X + 884.90355, where Y represents the RLS intensity of the scattering peak and X represents the concentration of Cys. The LOD was 64 nM. In Figure 7c, FL intensity increased linearly with the raised concentration of GSH, ranging from 0 to 200 μM, with an equation Y = −4.25229X + 824.12289, R^2^ = 0.99479. The LOD was 74 nM. In sum, with regard to both emission peak and scattering peak, the probe displayed an excellent linear for Cys and GSH detection. The calculation of the LOD uses the equation: LOD = 3σ/s; where σ is the standard deviation of the blank solution and s is the slope of the linear equation. The S/N ratio is 500:1 r.m.s.; however, as is shown in Table 1, compared with other methods in the literature, in our approach, by combining the fluorescent method and RLS technique, the detection sensitivity of RLS is much higher than that of the fluorescence method, achieving a lower detecting limit, as shown in Table 1. More importantly, we can quantitatively detect Cys by the fluorescent method, and detect GSH by the RLS technique, therefore, we can distinguish Cys and GSH in the same system, with the same equipment.

### 2.6. Application of C/N-Dots in FBS

Herein, to explore the practical application, this probe was applied to detect Cys in an FBS sample. The sample serum was purchased and kept at -20 °C, and then, after protein was removed, the serum sample was diluted 300-fold to ensure the Cys and GSH concentration fit the linear detection requirement, and was used to detect Cys and GSH, with different concentrations of Cys or GSH added to the probe solution. Then, the corresponding fluorescence responses of the probe at 430 nm (for Cys) and 345 nm (for GSH) in real samples were collected. Finally, the recovery was obtained; the recoveries of Cys are displayed in Table 2, ranging from 95.6% to 99.7%. Recoveries for GSH ranged from 97.3% to 102% in Table 3, indicating the detecting possibility in practical samples of this probe.

### 2.7. Application in Cellular Fluorescence Image

**Figure 8:**
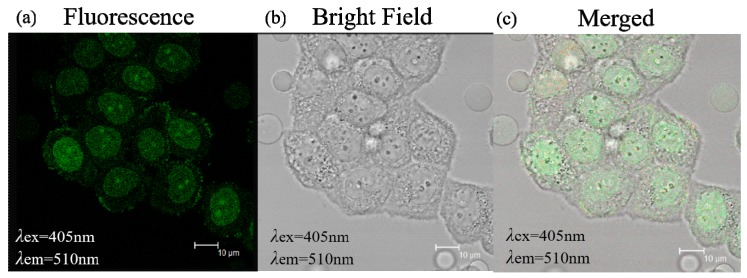
(**a**) Fluorescence microscope image of C/N-dots and the corresponding bright field transmission image (**b**) and merged image (**c**) of Hela-229 cells.

Due to their excellent optical properties, small size and good biocompatibility, C/N-dots can be used as an ideal fluorescent probe for qualitatively analyzing and quantitatively detecting specific substances with superior selectivity in bioimaging fields. Therefore, the application of C/N-dots for cellular fluorescence imaging was studied by using HeLa-229 cells. As is shown in Figure 8, under 405 nm exaction wavelength, the images of C/N-dots showed a green emission. The sample was observed in Leica SP2 confocal microscopy with excitation wavelength at 405 nm, and the cells emitted green fluorescence, proving that the C/N-dots effectively entered into the cells. More importantly, these results further proved the potential application of C/N-dots in cellular fluorescence imaging.

## 3. Mateials and Methods

### 3.1. Materials and Chemicals

Tartaric acid, ethylenediamine, and n-butyl alcohol were purchased from Aladdin Chemical Reagent Co. Ltd. (Shanghai, China), ZnSO_4_·7H_2_O, Co(NO_3_)_2_·6H_2_O, KNO_3_, AgNO_3_, Cr(NO_3_)_3_·9H_2_O, Ni(NO_3_)_2_·6H_2_O, Pb(NO_3_)_2_, MnCl_2_·4H_2_O, HgCl_2_, Cd(NO_3_)_2_·4H_2_O, FeCl_3_·6H_2_O, CuCl_2_·2H_2_O, NaCl, BaCl_2_·2H_2_O, MgCl_2_·6H_2_O, CaCl_2_, NaH_2_PO_4_·2H_2_O, Na_2_HPO4·12H_2_O, spectral pure KBr, Cysteine (Cys) Serine (Ser), Alanine (Ala), Glutamic acid (Glu), Arginine (Arg), Glutamine (Gln), Aspartic acid (Asp), Threonine (Thr), Methionine (Met), Isoleucine (Ile), Asparagine (Asn), Leucine (Leu), Histidine (His), Lysine (Lys), Valine (Val), Proline (Pro), Phenylalanine (Phe), Tryptophan (Trp), Glycine (Gly) and Glutathiose (GSH) were supplied from YongDa Chemical Reagent Co. Ltd. (Tianjin, China). HeLa-229 cells were obtained from the Cell Biology of Zhong Qiao Xin Zhou Cell Research (Shanghai, China). Fetal bovine serum (FBS) was bought from Shanghai sango biotechnology co., Ltd, CLARK, USA. Streptomycin and penicillin were bought from HyClone, Logan, USA. All the reagents mentioned above were of analytical reagent grade and used directly without further purification. The ultrapure water was used in all the experiments.

### 3.2. Apparatus

High-resolution transmission electron microscopy (HRTEM) images were taken on a FEI TF-20 microscope (FEI, Hillsboro, PerkinElmer, Boston, USA). The fluorescence spectra were carried out on an LS-55 fluorescence spectrometer (Nicolet Co., Madison, WI, USA). The Fourier transform infrared (FT-IR) spectrum was obtained with a Magna-IR560 FT-IR spectrometer (Nicolet Co., Madison, WI, USA) within the range of 400–4000 cm^−1^, while the UV-vis absorption spectra were performed on a UV-2550 spectrophotometer (Shimadzu, Kratos, Japan). The X-ray photoelectron spectroscopy (XPS) images were captured on an AXIS ULTRA DLD X-ray photoelectron spectrometer (Kratos, Manchester, UK). The fluorescence cell images were performed with a Leica SP2 confocal microscope.

### 3.3. Preparation of the C/N-Dots

A total of 3.0 g of tartaric acid was dissolved in 28 mL of ultrapure water in a 50 mL glass beaker under stirring. After the dissolution of the precipitation, 2 mL of ethylenediamine was added into the above solution, then the mixture was transferred into 50 mL Teflon-lined stainless steel reactor, and heated at 180 °C for 5 h, then the as-prepared C/N-dots solution was cooled down to room temperature in air, and the solution was purified with n-butyl alcohol. The resulting C/N-dots were stored away from light.

### 3.4. Cys and GSH Sensing

The detection of Cys and GSH was carried out in a phosphate buffer and at an excitation wavelength of 345 nm. The emission peak was located in 430 nm, and RLS peak was located in 345 nm. Not only the emission peak, but also the intensity of RLS, were studied. In order to investigate the optimal pH condition, solution with three different pH values were studied. An appropriate quantity of C/N-dots dispersion was added into 3 mL phosphate buffer, then, 100 μL (0.01 M) of Cys and GSH were added, respectively. The fluorescence emission spectra and RLS spectra were recorded.

### 3.5. Selectivity and Interference Measurements

The selectivity of Cys sensing was measured by adding other amino acid, such as Ser, Ala, Glu, Arg, Gln, Asp, Thr, Met, Ile, Asn, Leu, His, Lys, Val, Pro, Phe, Trp, Gly and GSH to replace Cys. An appropriate amount of C/N-dots were diluted in 3 mL phosphate buffer, then 30 μL amino acid was added, then the fluorescence emission spectra and RLS spectra were captured. The same detection conditions were applied for each section.

### 3.6. Detection of Cys and GSH in FBS

The serum sample contains protein, biothiols, etc. In order to simplify the detection condition, we removed the protein by mixing with ethyl alcohol at the radio of 1:2 to precipitate protein, keeping string for 2 min. The mixture was transferred into a centrifuge tube to centrifuge at 10,000 rpm for 30 min. Then, the precipitation was removed and the remaining solution was diluted at 300 folds to ensure the GSH concentration fit the linear detection requirement, and obtained recovery. The real-sample analysis of GSH in FBS serum indicated the feasibility and practicability of this probe.

### 3.7. Fluorescence Imaging

HeLa-229 cells were cultured in 1640 supplemented with 15% fetal bovine serum (FBS) (Shanghai sango biotechnology co., Ltd, CLARK, USA), 100 μg/mL streptomycin and 100 units/ml penicillin (HyClone, Logan, USA). The cells were digested with 0.25% Trypsin-EDTA, 1000 RPM centrifugal 5 min, after which we added 4 mL of medium in a humidified atmosphere with 5% CO2 at 37 ℃. Medium was replaced every 2–3 days. When confluence reached approximately 80%–90%, the C/N-dots were added to the cell culture, for another 7 h at 37 °C, to incubate. Then, the cells were rinsed with PBS at pH = 7 three times to eliminate the extra C/N-dots. Finally, the fluorescence images were captured under a Leica SP2 confocal microscope with excitation wavelength at 405 nm.

## 4. Conclusions

In summary, a new biosensing system was prepared by combining fluorescence analysis and RLS. A C/N-dots–Ag^+^ complex system was designed, in which an Ag^+^ complex provided RLS effect and C/N-dots provided the fluorescent detection. Herein, based on two different quenching mechanisms (the quenching of the fluorescence emission peak and the quenching of the RLS peak), two different methods were implemented to detect Cys and GSH in the same system. Quenching of fluorescence emission peak is suitable for selective detection, with high sensitivity, and ordinary operation. In the selective experiment on amino acids, Cys could be detected selectively, while other ammonic acids had no response. A good linear range and high sensitivity could be obtained when the sensitivity to Cys and GSH was detected by the effect of RLS. The two methods complemented each other and could selectively distinguish between Cys and GSH. With an LOD down to 13.9 μM and 222 nM for Cys and GSH, respectively, the sensing probe has been successfully applied for the analysis of them in an FBS sample.

## Figures and Tables

**Table 1:** Comparison of recently reported fluorescent CDs for detection of Cys and GSH.

Sample Detection	Linear Range (μM)	LOD (nM)	R^2^	Ref.
Cys	0.1–100	80	0.998	[51]
	1.0–110	160	0.998	[52]
	0–24	140	0.985	[53]
	0–167	64	0.997	This work
GSH	0.5–48	87	0.986/0.984	[54]
	1–10	300	0.997	[55]
	1–200	10	0.983	[56]
	0–200	74	0.995	This work

**Table 2:** Analytical results of Cys in FBS serum sample.

Sample	Added (μM)	Founded (μM)	Recovery (%)
Serum	0 16.7 150 167	16.2 46.7 166 179	- 95.6 99.7 98.0

**Table 3:** Analytical results of GSH in FBS serum sample.

Sample	Added (μM)	Founded (μM)	Recovery (%)
Serum	0 66.7 83.3 100	78.2 141 161 181	- 97.3 99.6 102
